# Healthcare Professionals’ Own Experiences of Domestic Violence and Abuse: A Meta-Analysis of Prevalence and Systematic Review of Risk Markers and Consequences

**DOI:** 10.1177/15248380211061771

**Published:** 2022-01-03

**Authors:** Sandi Dheensa, Elizabeth McLindon, Chelsea Spencer, Stephanie Pereira, Satya Shrestha, Elizabeth Emsley, Alison Gregory

**Affiliations:** 1 Domestic Violence and Abuse Health Research Group, Centre for Academic Primary Care, Bristol Medical School, 1980 University of Bristol, Bristol, UK; 2 The Royal Women’s Hospital, 2281 University of Melbourne, Melbourne, VIC, Australia; 3 5308 Department of General Practice, University of Melbourne, Melbourne. VIC, Australia; 4 5308 Kansas State University, Manhattan, KS, USA; 5 Departamento de Medicina Preventiva, Faculdade de Medicina, 28133 Universidade de São Paulo, São Paulo, Brazil; 6 92962 Kathmandu University School of Medical Sciences, Dhulikhel, Nepal; 7 Dhulikhel Hospital, 92961 Kathmandu University, Dhulikhel, Nepal

**Keywords:** Domestic Violence, Intimate Partner Violence, Health Personnel, Nurses, Physicians

## Abstract

**Background:** Globally, healthcare professionals (HCPs) are increasingly asked to identify and respond to domestic violence and abuse (DVA) among patients. However, their own experiences of DVA have been largely ignored.

**Aim:** To determine the prevalence of current and lifetime DVA victimisation among HCPs globally, and identify risk markers, consequences and support-seeking for DVA.

Method: PubMed, EMBASE, PsycINFO, CINAHL ASSIA and ProQuest were searched. Studies about HCPs’ personal experience of any type of DVA from any health service/country were included. Meta-analysis and narrative synthesis were adopted.

**Results:** Fifty-one reports were included. Pooled lifetime prevalence was 31.3% (95% CI [24.7%, 38.7%] *p* < .001)) and past-year prevalence was 10.4% (95% CI [5.8%, 17.9%] *p* <.001). Pooled lifetime prevalence significantly differed (Qb=6.96, *p* < .01) between men (14.8%) and women (41.8%), and between HCPs in low-middle income (64.0%) and high-income countries (20.7%) (Qb = 31.41, *p* <.001). Risk markers were similar to those in the general population, but aspects of the HCP role posed additional and unique risks/vulnerabilities. Direct and indirect consequences of DVA meant HCP-survivors were less able to work to their best ability. While HCP-survivors were more likely than other HCPs to identify and respond to DVA among patients, doing so could be distressing. HCP-survivors faced unique barriers to seeking support. Being unable to access support – which is crucial for leaving or ending relationships with abusive people – leaves HCP-survivors entrapped.

**Conclusion:** Specialised DVA interventions for HCPs are urgently needed, with adaptations for different groups and country settings. Future research should focus on developing interventions with HCP-survivors.

## Introduction

Domestic violence and abuse (DVA) includes physical, sexual, economic, psychological, emotional and other types of violence and/or abuse, as well as any controlling, coercive, violent or threatening behaviour, between people who are, or who have been, intimate partners or family members. The UK government defines DVA as being between people aged 16 and over (Domestic Abuse Act, 2021). Intimate partner violence (IPV) is the most reported type of DVA: the World Health Organization (WHO) has measured IPV in girls and women aged 15 and above and found that the physical and sexual forms affect 26%, with lifetime prevalence being particularly high in less developed countries (WHO, 2021). DVA can be lethal and is a human rights and a public health issue. The experience of DVA is gendered: for example, with regards to IPV, women victims are more highly victimised, injured and fearful than men (Hamberger & Larsen, 2015) and as Stöckl et al.’s (2013) review found, 38.6% of femicides are perpetrated by an intimate partner, compared with 6.3% of male homicides. Female survivors of DVA have a threefold risk of developing depressive disorders, a fourfold risk of anxiety disorders and a sevenfold risk of post-traumatic stress disorder (PTSD) (Chandan et al., 2020; Trevillion et al., 2012). DVA is associated with suicide and attempted suicide (Devries et al., 2011). Physical sequalae are numerous, including injury-related disability, HIV, chronic pain and gastro-intestinal conditions (Campbell, 2002; Garcia-Moreno et al., 2012).

Healthcare professionals (HCPs) are widely trusted and may be the first or only professionals to whom survivors disclose abuse (e.g. AIHW, 2019; Ansara & Hindin, 2010). Globally, HCPs are increasingly tasked with DVA enquiry, sensitive response focussing on risk and safety, and referral for onward support (WHO, 2017). While there is some research exploring HCPs’ attitudes and beliefs about DVA, and the impact of these on responses to patients (e.g. Sardinha & Nájera Catalán, 2018), HCPs’ own experiences of DVA have been largely ignored in research and interventions. Research about violence and abuse towards HCPs has focused almost exclusively on workplace violence, which affects 62% of HCPs globally (Liu et al., 2019) and is perpetrated mostly by patients (Mento et al., 2020). HCPs are a population worthy of enquiry regarding DVA, not least because in the majority of countries, health services are highly feminised workforces (e.g. Boniol et al., 2019; OECD, 2019). Despite the dearth of formal research about HCPs as DVA survivors, data from the United Kingdom (UK) indicates a high prevalence. A 2016 survey found that 14% of 2067 female nurses, midwives and healthcare assistants in England and Wales had experienced DVA in the past year, which is twice the prevalence as in the general population (Cavell Nurses’ Trust, 2016). Additionally, a UK 10-year femicide census showed that ‘HCP’ was one of the most commonly reported occupations of victims (Femicide Census, 2020).

In addition to its physical and mental health consequences, DVA can affect survivors’ performance and productivity at work (MacGregor et al., 2019, 2020; Oliver et al., 2019). Among HCPs, these outcomes may affect patient care: poor wellbeing and burnout are associated with self-reported medical errors (Baer et al., 2017; Wen et al., 2016). HCPs who are survivors may face a double burden, with the trauma and mental health sequelae of DVA compounding the challenging, stressful and potentially traumatic elements of healthcare jobs. DVA may affect work performance more directly if perpetrators interfere with or sabotage survivors’ work (MacGregor et al., 2019; Wathen et al., 2018).

Hernandez et al. (2016) conducted a review of studies, case examples and anecdotal evidence of physicians’ and medical students’ experiences of IPV and found relatively low prevalence among physicians across studies. However, the authors suggested that these studies may underestimate actual prevalence since stigma may prevent disclosure, and they emphasised a need for an improved understanding of physicians’ experiences of IPV. The review, while important, had several limitations: it focused solely on IPV, excluding other types of DVA; it focused on physicians solely, identifying only four research studies with participants who had experienced IPV in adulthood rather than being exposed to IPV in childhood; and it did not use meta-analysis to calculate pooled prevalence. The aim of the systematic review reported in the present paper was to extend and broaden the work of Hernandez and colleagues by conducting a mixed-methods systematic review including meta-analyses to investigate DVA experiences among all groups of HCPs. The specific objectives were to (1) conduct a meta-analysis to determine the pooled prevalence of current and lifetime DVA among HCPs; (2) explore risk markers; (3) explore the effects of DVA on physical and mental health and on work performance (including responding to survivor patients); (4) explore the support HCPs want, seek and receive.

## Methods

This review is reported in line with PRISMA guidelines (Page et al., 2021). The protocol is registered in Open Science Framework (Dheensa et al., 2020).

### Information Sources and Search Strategy

**Figure 1.**
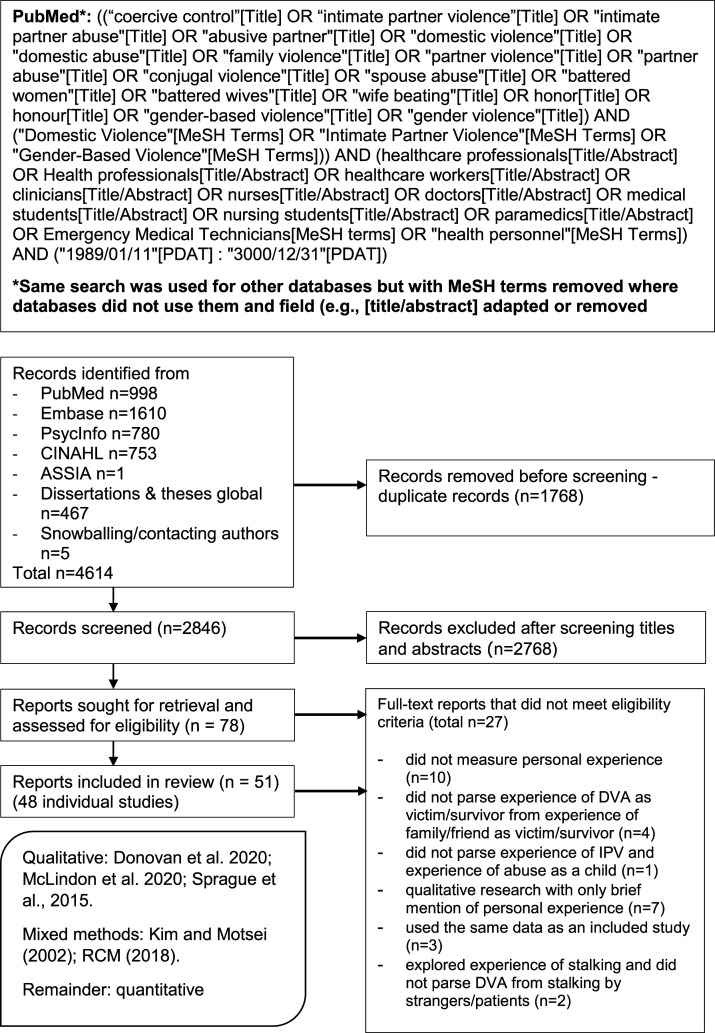
Search strategy and PRISMA flow diagram.

The search strategy (Figure 1) was developed in collaboration with an information specialist by adopting DVA-relevant terms from recent systematic reviews (e.g. Hawcroft et al., 2019). We combined these, using Boolean logic, with terms relating to healthcare professions. Syntax and spelling were reviewed. Peer-reviewed and grey literature reports of primary studies published from January 1989 were sought. The searches were conducted on September 17, 2020 and updated on May 15, 2021 within PubMed, EMBASE and PsycINFO (via Ovid), CINAHL (via EBSCOhost) and ASSIA and Dissertations and Theses Global (via ProQuest). MeSH subject headings were used where appropriate. We also snowball searched using Google Scholar and did a general Google search for non-academic reports. We contacted recent reports’ authors to identify further articles of interest.

### Eligibility Criteria

Studies about any HCPs in any job roles, in any parts of a health service/system, in any country, and about personal experiences of any type of DVA (i.e. between intimate partners and family members) were included. Primary outcomes of interest were lifetime and past-year prevalence of DVA victimisation. We chose not to include studies with a specific focus on child abuse, although it can overlap with DVA: for example, we included studies that measured IPV in girls and women aged 15+. To give the review boundaries, we excluded reports of qualitative studies where HCPs’ own experiences of DVA were not the main topic or a key theme (e.g. Mezey et al., 2003), and where HCP and administrative staff participants were not distinguished, whether in a mixed professional study (e.g. Giesbrecht, 2020a) or healthcare staff-only study (Rothman et al., 2007). We excluded a mixed-methods study whose quantitative sample likely overlapped with that of Cavell Nurses Trust (2016) and qualitative analysis was unclear on which participants were survivors (McGregor et al., 2016). Reports were also excluded if all participants were students (e.g. Frank et al., 2006); it was unclear whether the study definition of ‘personal experience’ related to primary experiences of DVA in adulthood or secondary exposure to DVA through, for example, a friend or family member (e.g. Beynon et al., 2012; Dickson & Tutty, 1996; Moore et al., 1998; Sis Çelik & Aydın, 2019); the study looked at experiences of child abuse or childhood exposure to DVA in the household only (e.g. Haj-Yahia et al., 2009); or if the study separately measured experiences of IPV and child abuse but reported them as a composite outcome measure (e.g. Sugg & Inui, 1992); We did, however, include two studies (McLindon et al., 2018; Siltala et al., 2019) that measured lifetime violence/abuse from family members where respondents were not asked at what age they experienced abuse, so figures may include childhood abuse. Finally, we excluded reports if they were in a language unknown to authors and not translatable using Google Translate.

### Selection Process, Data Collection Process, and Data Items

Search results were exported into EndNote X9 and de-duplicated. SD and a research assistant (RA) independently examined titles and abstracts to exclude any clearly irrelevant results. With the remaining reports, SD and the RA independently screened the full texts, documenting those that met the eligibility criteria and resolving any disagreements about eligibility through discussion. Decisions regarding articles that initially met the criteria, but were later excluded, were documented – these included ‘duplicate’ reports of the same study with minimal difference (Carmona-Torres et al., 2017, 2019). SP screened one Spanish-language full text. Two Turkish abstracts (Hotun Şahin et al., 2008; Kiyak & Akin, 2010) were excluded as Google translations were incomprehensible.

For quantitative studies, SD developed a data extraction spreadsheet in Microsoft Excel and extracted data from each article (aside from one Spanish language article, for which SP extracted the data). (EM, SP, SS, EE and AG) and the RA each checked the data extraction for a subset of the English-language reports, so each report was double-checked. Where information in a report was unclear, SD contacted the study authors for clarification. SD and the second reviewer for each report resolved any discrepancies through discussion. The data extraction form was piloted and revised iteratively. The primary data extracted were on lifetime and past-year prevalence of DVA. Additional data on study characteristics, DVA subtype, gender of HCP-survivors, demographic information (household income, education level, ethnicity, age and children), the impact of experiences on health and work and support seeking were collated. Where a report did not specify participant gender at all, we assumed all participants were women.

Based on the quantitative results and an initial reading of qualitative reports, SD created a framework in Microsoft Word to capture relevant qualitative data (Dixon-Woods, 2011). The themes in the framework related to DVA risk, protective factors and barriers to accessing community and workplace support. SD, SP and SS coded the qualitative findings, extracting the coded data into the framework. AG performed confirmatory double-coding, reframing and honing the themes as necessary.

### Risk of Bias Assessment and Critical Appraisal of Quality

We assessed the risk of bias within quantitative studies using the Hoy et al. (2012) 10-item checklist and, as per previous research (e.g. Patel & Roesch, 2020), used numerical scoring. We weighted each item equally: studies were scored low risk (scoring a 0) or high risk (scoring a 1) for each of the 10 items. A summary score indicated the overall risk of bias, categorised as low (0–3), moderate (4–6), or high (7+). We also explored the risk of bias across studies (e.g. language bias, location bias) (Boutron et al., 2021). Qualitative reports were appraised using Critical Appraisal Skills Programme (CASP, 2018) tools: these are checklists designed for use in different types of research study.

### Synthesis Methods and Effect Measures

Using Comprehensive Meta-Analysis 3.0 (Borenstein et al., 2013), meta-analyses of data from quantitative studies on lifetime and past-year experience of DVA were conducted to calculate pooled prevalence figures (%). Heterogeneity was found among studies (I^2^ = 98.75), and we used a random-effects approach to account for between-study and within-study differences (Card, 2012). Forest plots were created to display the data. Additionally, subgroup analyses and Q-statistics were calculated to determine if there was significant heterogeneity in lifetime DVA between men and women, between nurses and physicians and between low to middle-income countries (LMICs) and high-income countries (HICs), since DVA disproportionally affects women, particularly in LMICs (WHO, 2021), and a higher proportion of nurses, compared with physicians, are likely to be women. Subgroup analyses used data from subsets of participants within each study, where available, and thus studies were included in more than one subgroup where appropriate. Studies that did not parse data by gender/profession were not included in the respective subgroup analyses.

Measures used across studies (e.g. demographics, impact of DVA on health and work) were heterogeneous, both in terms of what specifically was measured and how. A narrative synthesis was thus employed (Popay et al., 2006) to present these quantitative results. Test statistics from original articles are presented if an association was found: since studies reported different test statistics (e.g. *p* values, percentages, CIs), our reporting of these statistics varies according to the data each article presents. Qualitative findings were integrated with the quantitative results, and relevant quotations and the person’s profession (in brackets) are used for illustration.

## Results

### Study Characteristics

Of 2846 articles identified and screened, 51 articles were ultimately included – 46 quantitative, three qualitative and two mixed methods – reporting on 48 studies. Three articles presented data from studies reported in another article (Al-Natour, Gillespie, Wang, et al., 2014; McLindon et al., 2018, 2019). The PRISMA flow diagram is shown in Figure 1. The articles we included were published between 1993 and 2021. Study populations came from 19 countries, predominantly the United States (US) (19/48), followed by South Africa (4/48) and the UK (4/48). The sample included in the review was therefore somewhat diverse in terms of race, ethnicity, language, nationality, geography and culture. The quantitative dataset contained a combined sample of 25,111 participants who answered questions about DVA experiences. Of these, approximately 11,440 were nursing and midwifery staff, 8538 were doctors/physicians, 882 were surgeons, 536 were emergency medicine staff, 95 were psychologists/mental health staff and the remainder were a mixture of unspecified professions. The sample was not diverse in terms of gender identity. While an exact breakdown of gender is not possible, approximately 21,594 were women, 3465 were men, 7 were trans/non-binary and 87 were unspecified. Twenty-eight studies exclusively included women. Most studies explored IPV only, but 17 also explored other types of DVA, that is, between adult family members. Quantitative study sample sizes ranged from 28 (Sawyer et al., 2018) to 4501 (Doyle et al., 1999) and qualitative study samples sizes from 20 (Sprague et al., 2016) to 93 (McLindon et al., 2020). Guzmán-Rodríguez et al. (2019) was the only study to measure DVA longitudinally: time 2 DVA data were used for analysis. Online Supplemental Appendix A (Table) contains study details.

### Risk of Bias and Critical Appraisal

Risk of bias within quantitative studies was moderate for 10 studies and low for all others (mean=2.36, indicating a relatively low risk of bias overall). Online Supplemental Appendix B (Table) contains detailed scoring. Common areas of bias were non-randomised sampling, low response rate (<65%, Kelley et al., 2003), use of measures that were non-validated (e.g. a question like ‘have you ever been a victim of domestic violence’ rather than a validated measure such as the Composite Abuse Scale) and/or failed to encompass non-physical DVA. Regarding risk of bias across studies, safeguards to prevent non-reporting biases (e.g. study registration) are not routinely adopted in this topic area, so publication bias is likely. Bias is likely also because non-significant findings from countries where English is not the first language are less likely to be published in English language journals (Egger et al., 1997). All qualitative research articles were judged to be of good quality (CASP, 2018) although reflexive analysis was often missing.

### Prevalence of Domestic Violence and Abuse among Healthcare Professionals

Online Supplemental Appendix A (Table) contains a summary of prevalence data from each study. The pooled lifetime prevalence of DVA victimisation for HCPs from 38 studies was 31.3% (95% CI [24.7%, 38.7%] *p* < .001). The pooled past-year prevalence of DVA victimisation for HCPs from 11 studies was 10.4% (95% CI [5.8%, 17.9%] *p* < .001). Using subgroup analysis, we found that pooled lifetime prevalence significantly differed (Q^b^=6.96, *p* < .01) between male (14.8%) and female (41.8%) HCPs, and by clinical profession: specifically between nurses (35.4%) and physicians (12.1%) (Q^b^=10.51, *p* < .01). Pooled lifetime prevalence also differed between LMICs (64.0%) and HICs (20.7%) (Q^b^ = 31.41, *p* < .001). Online Supplemental Appendix C contains the forest plots.

Only two articles reported current/past 12 months DVA categorised by gender: both explored IPV only, and in both, higher percentages of men than women were affected. Mitchell et al. (2013) found that a higher percentage of men (9%, 4/45) than women (8.2% (24/294) had been hit, slapped, kicked or physically hurt. Cavell Nurses Trust (2016) found that a higher percentage of men than women had experienced non-physical abuse, for example, prevented from having money or belittled (15%, 15/100 men vs. 12%, 186/1546 women) and ‘force’, for example, pushed, slapped, hit or punched (5%, 5/100 men vs. 3%, 46/1546 women). However, the Trust also found that more women (4.6%, 71/1546) than men (2%, 2/100) had felt frightened or threatened by their partner in the last 12 months. Neither of the studies conducted statistical comparison tests, and as the Trust points out, the male sample was much smaller than the female sample thus increasing the possibility of statistical error.

Comparing subtypes, in all studies that measured non-physical forms of DVA, this subtype was more prevalent than physical/sexual DVA (see Online Supplemental Appendix A Table).

### Perpetration of Domestic Violence and Abuse by Healthcare Professionals

Five articles reported findings about perpetration by HCPs. In two articles that explored gender differences, more women than men disclosed using abusive behaviours. Early and Williams (2002) found that 22.6% (44/195) of participants had perpetrated physical DVA: 25.6% (41/160) of women and 8.6% (3/35) of men. The overlap between those who experienced and used physical DVA was not reported. Similarly, Mitchell et al. (2013) reported that 21% (75/357) of participants had perpetrated physical DVA – 21.4% (63/294) of women, 15.6% (7/45) of men and 27.8% (5/18) of those whose gender was unspecified. Of those who disclosed perpetration, 81.3% (61/75) had also experienced DVA, possibly suggesting bidirectional violence. Kim and Motsei (2002) asked their eight male participants about abusive behaviours towards a partner: six had been abusive, and of these, four had used physical abuse, three had been sexually abusive and six had used emotional abuse. Finally, two studies asked HCP-survivors if the perpetrator was also a HCP. Acquadro Maran and Varetto (2018) found that of 147 HCPs who had experienced DVA and stalking, another HCP, mostly an ex-partner, was the stalker in 41 cases. A gendered breakdown of HCP-stalkers was not provided, but overall figures showed that most stalkers were men. Donovan et al. (2020) found for 7/21 female doctor-survivors, the perpetrator was also a doctor.

### Demographic and Job-related Risk Markers for Domestic Violence and Abuse among Healthcare Professionals

Online Supplemental Appendix D (Table) contains details (including statistics as reported in studies) on risk markers. As well as being female, a nurse or from a LMIC, the following risk markers were associated with experiences of DVA: ethnicity (though no clear pattern emerged about the ethnic groups most at risk) (Bracken et al., 2010; Doyle et al., 1999; Reibling et al., 2020); increasing number of, or having no, children (Aguocha et al., 2017; Shokre & Ahmed, 2017) (although Çakmak Pekşen and Şahin (2019) found number of children was not related) and financial factors. Specifically, women who were dependent solely on their own salary, women whose husbands were unemployed and women who contributed a greater share to household expenses were more likely to experience DVA (Carmona-Torres et al., 2018; Sharma & Vatsa, 2011; Shokre & Ahmed, 2017) – although Bracken et al. (2010) found no association with income itself. Education was also related: qualifications were inversely associated with experience of DVA (Aguocha et al., 2017; Bracken et al., 2010; Khan et al., 2014; Sharma & Vatsa, 2011). However, four studies (Díaz-Olavarrieta et al., 2001; Guzmán-Rodríguez et al., 2019; La Flair et al., 2012; Mariano, 2001) found no association with education level and LaFlair also found no association with ethnicity. Doyle et al. (1999) and Stein et al. (2021) were the only studies to explore the relationship between DVA and sexual orientation: neither study reported it as significant in regression analyses. Both found that a history of mental health problems was also a risk marker for DVA. In total, 17 quantitative studies explored these risk markers and 13 found an association.

Qualitative findings highlighted additional risk markers and suggested that some aspects of being a HCP made people especially vulnerable to DVA. For example, geographical relocation for medical jobs led to social isolation among doctor-survivors (Donovan et al., 2020). In addition, nurse-survivors in Kim and Motsei (2002)’s study commented that their husbands felt threatened by their income-earning and professional status. Their husbands also accused them of sexual infidelity with male colleagues and assaulted them for interacting with these colleagues. Moreover, since many of the nurses had been financially dependent on their husbands during nursing training, the husbands claimed this as a justification for controlling the money they now earned. In another study (Donovan et al., 2020), female doctor-survivors reflected that personal characteristics associated with being a doctor (compassion, tolerating abuse and a culture of thanklessness from colleagues and patients) may have led them to tolerate abuse at home; ‘the culture at work, where we don’t get thanked and we get…bullied quite a lot, just sets you up to think that’s normal at home as well’ (doctor, Donovan et al., 2020, p.195).

### Impact on Physical, Reproductive, and Mental Health

Eleven quantitative studies explored and found associations between DVA and health consequences, which were acute and chronic. DVA caused a range of direct physical health consequences. Nurse-survivors had been directly injured as a result of physical or sexual DVA: 2.2% (36/1646) in the Cavell Nurses Trust’s (2016) study and 40% (12/30) in Sharma and Vatsa’s (2011) study, where notably, only three received any care for injuries. Shokre and Ahmed (2017) found, similarly, nurses had accidents at home with delayed treatment (42.3%, 137/324) and recurrent head/neck injuries (11.7%, 38/324). Physician-survivors too were more likely to have been hospitalised than physicians who had not experienced DVA (11% vs. 3.4%, *p* < .01) (Doyle et al., 1999). However, it is unclear from these two studies whether the accidents, injuries and hospitalisations were unrelated to, or caused directly or indirectly by, DVA. Regarding indirect physical health consequences, physician-survivors had 3.2× greater odds of having chronic fatigue syndrome than those who had not experienced DVA (Doyle et al., 1999). Nurse-survivors had higher miscarriage rates than those who had not experienced DVA (41% vs. 15%) (Selek et al., 2012) and high preterm labour rates (18.2% [59/324]) (Shokre & Ahmed, 2017). HCP-survivors scored worse on sleep quality (*p* = .001) (Siltala et al., 2019), and experienced sleep disorder (44.2%, 65/147), headaches (27.2%, 40/147), weakness (22.5%, 33/147), stomach trouble (18.4%, 27/147), weight changes (17.7%, 26/147) and nausea (8.8%, 13/147) (Acquadro Maran & Varetto, 2018). Mental health consequences were common among all HCPs: for example, severe stress, depression, panic attacks (19.7%, [29/147] Acquadro Maran & Varetto, 2018) and suicide ideation and attempts (Doyle et al., 1999). Online Supplemental Appendix D details mental health outcomes (including statistics as reported in studies).

### Links to Workplace Violence

Three studies highlighted an overlap between experiences of DVA and workplace violence. Early and Williams (2002) found that 19.5% (38/195) of nurse-survivors had been physically assaulted by their partner and by a patient. An even higher percentage was found by Oliveira and D'Oliveira (2008) who explored violence from patients and colleagues: 46.4% (52/112) of nurse-survivors experienced psychological (mainly from male colleagues and bosses) and physical (mainly from patients and their companions) workplace violence. This violence not only affected nurses: McLindon et al.'s (2020) qualitative research showed that a range of HCP-survivors had been victims of abuse from patients and bullying/harassment from colleagues. These experiences triggered traumatic memories of DVA experiences.

### Impact of Domestic Violence and Abuse on Work

Four quantitative studies explored and found that DVA affected work. Perpetrators directly interfered with nurse-survivors’ work – harassing them at work, lying to co-workers about them, sabotaging their transport to work, stealing transport money and restraining or threatening them to stop them going to work (Al-Modallal et al., 2016). The mental and physical health consequences of DVA also harmed survivors’ work lives. McLindon et al. (2020), although a qualitative study, found 60.8% of HCP-survivors reported that a physical or psychological injury directly affected them at work. DVA was linked to high rates of severe daily stress at work (*p* < .05) among physicians (Doyle et al., 1999). Among nurses, around half of survivors felt less able to concentrate on their work (56.7%, Sharma & Vatsa, 2011; 44.3%, 62/140, Al-Modallal et al., 2016) and were unable to work to the best of their ability (47.14%, 66/140, Al-Modallal et al., 2016). Around a quarter lost confidence in their own abilities (26.7%), and many took sick leave (23.3%, Sharma & Vatsa, 2011; 44.3% 62/140, Al-Modallal et al., 2016). In fact, around a third of nurse-survivors ended up quitting (32.86%, 46/140) or losing their job (31.4%, 44/140) due to DVA (Al-Modallal et al., 2016). Regarding job satisfaction, there was a strong inverse correlation with incidence of DVA (r = −0.62, *p* < .001) among nurses (Ortlepp & Nkosi, 1993). Similarly, Doyle et al. (1999) found physician experiences of DVA were associated with lower career satisfaction (*p* < .01).

Within the qualitative studies, HCP-survivors talked about taking sick leave because of DVA. Some felt unable to take time off, but at the same time, worried about going to work and making mistakes that might harm patients and cost them their job. Indeed, one midwife reported, ‘The stress at home resulted in me making a mistake at work which resulted in a full management investigation during which I was subjected to stress I had never known it was possible to endure’ (RCM, 2018, p.19). HCP-survivors reported that while struggling to work at full capacity, senior staff had given them negative appraisals, which they felt had affected their career progression and professional standing: ‘I tried to muddle through but (my supervisor) gave me a terrible report: “you’re not engaging in ward rounds; you’re not really talking to patients”’ (doctor, Donovan et al., 2020, p.197).

### Impact on Response to Domestic Violence and Abuse Among Patients

Nine quantitative studies explored the impact of being a survivor on response to DVA among patients. Although four studies found no differences between HCPs with and without personal experience of DVA, specifically on screening (Al-Modallal et al., 2016; Rodriguez et al., 1999), identifying (Price, 1999) and responding to patients experiencing DVA (Early & Williams, 2002), five studies did report differences. HCP-survivors identified more cases of DVA overall (46.6% vs. 30.3%, *p* < .01, Hinderliter, 2000). Nurse-survivors were more likely to report seeing patients who were survivors (χ2 = 19.86, *p* < .001, 86.8% vs. 13.2%, Christofides & Silo, 2005) and to ask direct questions about DVA (45% vs. 35%, *p* =.033, Stenson & Heimer, 2008; adj OR 1.5, 95% CI [1.0, 2.3], *p* = .07, McLindon et al., 2019). HCP-survivors were twice as likely to show sensitive attitudes towards patient-survivors (McLindon et al., 2019), and were more likely to access DVA information for them (OR =2.0, 95% CI [1.0, 4.0], *p* = .04, McLindon et al., 2019), refer them to a social worker, and protect their privacy and safety ( $\overset{-}{\text{x}}\;10.8\text{ vs}.\overset{-}{\text{x}}\;9{.1\text{, Wald χ}}^{2}=\;6.85\text{, }p\;=\;.03$ , Christofides & Silo, 2005). HCP-survivors also had a greater belief in their responsibility to respond to DVA than other HCPs (Lai, 2007).

Qualitative findings indicated that HCP-survivors described feeling able to respond to patient-survivors with more empathy, a better understanding of trauma (Donovan et al., 2020; McLindon et al., 2020), and even advice: ‘If you’ve found a solution to your problem you might share it with the patient [saying] “Sometimes I’ve had the same problem, this is what I did”’ (nurse, Sprague et al., 2016, p.177). Doctor-survivors moreover expressed how their own experiences had challenged their views about ‘typical DVA victims’, making them more likely to recognise that DVA could affect any one of their patients (Donovan et al., 2020). Kim and Motsei (2002) notably found that DVA training made HCPs acknowledge their own abuse experiences. The realisation was painful, but it led to them ‘speak[ing] out about’ it, which was ‘healing’ (nurse, p. 1248). However some HCPs, including those whose DVA experiences were more recent, found that working with patient-survivors – and in the hospital environment more generally – could be distressing: ‘too many things were hitting too close to home…I would just shut down’ (doctor, Donovan et al., 2020, p.197, p.197).

### Support

#### Proportion of Healthcare Professionals-Survivors Who Sought Support and from Whom

Five quantitative studies explored support-seeking. The proportion of HCP-survivors who sought support varied, from two-thirds among surgeons (Stein et al., 2021) to around a quarter (Carmona-Torres et al., 2018; Oliveira & D'Oliveira, 2008) among mixed professionals and nurses. Regarding the source of this support, Acquadro Maran and Varetto (2018) found that 51.7% (76/147) of HCP-survivors sought support from friends, family and neighbours. Aguocha et al. (2017) and Stein et al. (2021) similarly found that HCPs reached out predominantly for informal support. Use of formal legal support was rare: Selek et al. (2012) found that none of the 22 nurse-survivors took legal steps, and Stein et al. (2021) found that just 7.3% (21/287) of surgeons did so. Oliveira and D'Oliveira (2008) on the other hand found that HCP-survivors sought more formal support (from police and psychologists) than informal support, while (Carmona-Torres et al 2018) found a fairly even spread between informal and formal sources.

#### Experiences of Seeking Formal Support from Specialists and Professionals

Qualitative research showed that HCP-survivors, specifically midwives and doctors, reported positive experiences of seeking specialist DVA support (Donovan et al., 2020; RCM, 2018), counselling (RCM, 2018) and support from their own general practitioners (GPs) (Donovan et al., 2020). One doctor-survivor, however, indicated that her GP had not believed her – the survivor did not fit the GP’s misconception of a legitimate ‘victim’. Other doctor-survivors indicated that the professional to whom they disclosed had threatened to alert the survivor’s professional regulator. Doctor-survivors additionally felt that when the perpetrator was a doctor too, other professionals did not (seem to) believe he was abusive: ‘GPs and consultants, [were] standing up in court and saying they believe he couldn’t have done any of these things, how great he is’ (doctor, Donovan et al., 2020, p.196).

#### Experiences of Seeking Support from the Workplace

Although a few HCPs (Donovan et al., 2020; RCM, 2018) gave examples of asking for *and* receiving helpful support from the workplace (including flexible working to accommodate court cases, overnight stays on a hospital ward for safety and emotional support from senior staff), in general, seeking and receiving such support was uncommon. For example, Oliveira and D'Oliveira (2008) found just one participant (out of 179) had sought workplace-based support. RCM (2018) found that although a third (30%, 60/180) of midwife-survivors sought workplace support, only half of these received it. Overall, the findings from qualitative research indicated that HCP-survivors had had negative experiences of seeking workplace-based support: many relayed experiences where requests (e.g. adaptation to shift patterns) were denied. Worse still, several midwives reported that senior colleagues to whom they disclosed pressured them to report the DVA and return to work, alerted social services about their child without consent, and informed their professional regulator: ‘I was treated like the perpetrator. I was reported to [the regulator] for fitness to practice, yet I have a 100% clean work record over my long career’ (midwife, RCM, 2018, p.28). Doctors and midwives who had taken sick leave due to DVA felt forced to explain themselves and felt unsupported upon returning to work. In fact, some midwives reported that disclosure to senior colleagues made them feel more stigmatised and alone, and like a ‘nuisance’ (Donovan et al., 2020; RCM, 2018).

#### Reasons Healthcare Professionals-Survivors did not Seek Support

Of the quantitative studies, only Oliveira and D'Oliveira (2008) explored HCP-survivors’ reasons for not seeking support. These reasons were primarily ‘fear of exposure’, feeling ashamed, discouragement by close relatives, fear the perpetrator would retaliate and lack of time and financial resources. Qualitative findings similarly highlighted shame, stigma (Donovan et al., 2020; Kim & Motsei, 2002; RCM, 2018; Sprague et al., 2016) and a feeling that ‘it should not happen to us’ as barriers: ‘I felt ashamed that as a midwife I was experiencing it’ (midwife, RCM, 2018, p.16); ‘We’re not supposed to be vulnerable; we are supposed to be intelligent, strong women…“you should have known better”’ (doctor, Donovan et al., 2020, p.195). Doctor-survivors felt their income made them less vulnerable than other survivors, and so less deserving of professional support (Donovan et al., 2020).

In addition to the above, there were additional and specific reasons for not seeking workplace-based support, which were similar across studies. Nurse-survivors experienced a workplace culture of blame, gossip and ridicule (Sprague et al., 2016), and doctor-survivors reported that their medical colleagues were judgemental (Donovan et al., 2020). Nurse and midwife-survivors (RCM, 2018; Sprague et al., 2016) did not feel any sense of privacy and confidentiality at work, which presented a barrier to accessing peer and formal support (e.g. through employee assistance programmes) at work: All worried that disclosures would be used against them, or would affect their professional standing, registration with their regulator and career progression: ‘I would not want my workplace to know as it would impact upon my [registration]’ (midwife, RCM, 2018 p.20). On reasons for not seeking professional support outside of the workplace, doctor-survivors worried they might see their patients at DVA support groups. Others felt unable to seek such support due to feeling unable to take time off work (Donovan et al., 2020).

#### Support Survivors Wanted from Workplaces

Overall, HCP-survivors wanted their workplaces to be open, supportive and flexible to their needs, with an increased understanding among colleagues of staff experiences of DVA. Survivors wanted acknowledgement that they may not be able to work to the best of their ability, without fearing that they might lose their jobs. Survivors wanted sensitive support from qualified professionals (e.g. counsellors), as well as DVA-trained human resources staff and senior colleagues, with a guarantee of confidentiality. Additionally, survivors wanted support options, such as leave and flexible working, to be made more obvious, and to be formalised in policy (Donovan et al., 2020; McLindon et al., 2020; RCM, 2018).

## Discussion

This paper describes the first meta-analysis of data on prevalence, and systematic review on risk markers, consequences and outcomes of DVA among HCPs. Table 1 summarises the critical findings. Our results show a 31.3%. pooled lifetime prevalence of DVA victimisation among HCPs. This pooled prevalence differed significantly by gender (14.8% among men and 41.8% among women) and by profession (35.4% among nurses and 12.1% among physicians). Pooled lifetime prevalence of DVA victimisation also significantly differed between country settings and was especially high in LMICs (64.0%) compared with HICs (20.7%). The pooled past-year prevalence of DVA victimisation for HCPs was 10.4%. Non-physical abuse was more prevalent than physical/sexual abuse but was not always measured. Research by the WHO (2021) has found that 26% of ever-married/partnered women aged 15 years and older in the general population have experienced lifetime physical or sexual IPV. While these figures are not directly comparable to our findings (because DVA, not just physical/sexual IPV, was explored in our study), the prevalence for female HCPs remains comparably high. Several of the included studies stated that the prevalence they found was higher than that found in national surveys conducted with their country’s population (Bracken et al., 2010; Carlisle-Cohen, 1996; Carmona-Torres et al., 2018; Cavell Nurses’ Trust, 2016; Hinderliter, 2000; Janssen et al., 1998; Khan et al., 2014; McLindon et al., 2018; Ralph, 2000). In some cases, HCPs were poly-victimised by DVA and workplace violence. Our findings also demonstrate evidence for DVA perpetration by HCPs, including physical violence from women. When women use physical violence, it tends to be in response to violence initiated against them (Hamberger & Larsen, 2015), however studies did not explore the frequency, type, context (e.g. uni- or bidirectional, resistance, retaliation) or severity of perpetration. Notably, no research explored domestic homicides of HCPs, suggesting a need for further research.

**Table 1.** Critical findings: healthcare professionals’ (HCPs) experience of domestic violence and abuse (DVA).

1	Pooled prevalence of DVA victimisation for HCPs was 31.3% (lifetime) and 10.4% (past-year). Prevalence for female HCPs (41.8%), nurses (35.4%) and HCPs in low-middle income countries (64.0%) was especially high
2	DVA perpetration by HCPs was reported in some articles
3	Risk markers for DVA included ethnicity, number of children, financial factors, education and mental health history. Aspects of the HCP-role also made people vulnerable, for example, social isolation due to relocation and tolerating abuse from patients
4	Physical, indirect physical and mental health consequences of DVA were varied and common, and acute and chronic, and included suicide ideation
5	Many HCPs were victims of workplace violence from patients/colleagues as well as DVA
6	Perpetrators directly interfered with survivors’ work. DVA health consequences affected ability to work, concentration and confidence; led to making mistakes with patients; damaged career progression and professional standing; and led some survivors to quit
7	HCP-survivors were more likely to identify and respond sensitively and empathically to patient-survivors than HCPs without DVA experiences, but some found this distressing
8	Many HCP-survivors did not seek support at all. Those who sought workplace-based support reported harmful experiences, for example, having their professional regulator informed. Barriers to seeking support included fear of judgement, fear that disclosures would cast doubt on their fitness to practise, stigma and shame (‘we should know better’)
9	Survivors wanted workplace support that was flexible to their needs, with options for professional support (e.g. from counsellors) and for these to be formalised in policy. They also wanted colleagues to understand the impact of DVA, for example, on work ability

Risk markers (education, ethnicity, finances and number of children) and impact of DVA on physical and mental health were similar to those found in the general population (e.g. Capaldi et al., 2012; Garcia-Moreno et al., 2012; Yakubovich et al., 2018). This finding is important in healthcare settings because the ill-health of HCPs has the potential to impact detrimentally on patient care (Baer et al., 2017; Wen et al., 2016). DVA affected HCPs’ ability to work well (e.g. damaging their ability to concentrate and wellbeing), which echoes findings from related research in other job sectors: for example, Giesbrecht et al. (2020b) found 82.8% of survivors were unable to concentrate at work and 74% were unable to perform to best of their ability. As research from other job sectors shows (MacGregor et al., 2019; Wathen et al., 2018), DVA perpetrators sometimes directly interfered with survivors’ work and DVA caused some survivors to leave their profession entirely. A notable risk marker for DVA that emerged from LMIC settings was household finances: specifically female HCPs were more likely to be survivors if they were dependent solely on their own salary, contributed more money to household expenses and had unemployed husbands. When female HCPs had financially depended on their husbands to attend school, male partners claimed this as justification for controlling their subsequent income. These results may indicate that DVA against female HCPs in some LMIC settings includes financial abuse whereby men force women to be the main financial contributor and/or may indicate that DVA is linked to men’s feelings of disempowerment if they do not perceive themselves as the ‘breadwinner’. So, although women who do not work outside the home may face additional risks and fewer protective factors than women in paid work, being employed can introduce new challenges. This aligns with findings from MacGregor et al. (2020): while women experienced benefits from being in work – a sense of identity, agency, social support, escape from the violence and financial independence – they also felt unable to access services due to work, had to hide DVA at work, and felt perpetrators posed a danger to colleagues, among other challenges.

The reasons for the high prevalence among HCPs, and specifically among female HCPs, are unclear from the included studies. One potential explanation is that people with histories of trauma may be drawn to enter caring and altruistic professions, such as healthcare, where they can ‘give back’ and care for others who have experienced trauma. The literature describes such individuals as ‘wounded healers’, and evidence for this explanation exists within social work (Zosky, 2013), psychotherapy (Radu, 2013) and DVA specialist support services (Gilbert, 2020). A second potential reason is that the context and culture of their work, in particular the common occurrence of abusive behaviours within the workplace, may make it harder for HCPs to identify their experiences outside of work as DVA, and to leave or end a relationship with a person who is, or is becoming, abusive. Related to these two reasons, McGregor et al. (2016) suggest that people with caring, nurturing and protective traits may be drawn to healthcare, but these traits may leave them vulnerable to abusers. A third possibility is that if HCPs are in a relationship with a person who is becoming, or has become, abusive, the unique barriers HCPs experience, as identified by our review, prevent them from seeking informal and formal support. These barriers relate to HCP-survivors’ feelings around shame and judgement (in particular, that DVA should not happen to HCPs), practical obstacles relating to time and money (not having enough of either, or earning too much to feel ‘worthy’ of help), risks around lack of confidentiality (from colleagues and patients, e.g. being seen at DVA support services), concerns about disclosure affecting their work status (e.g. professional regulators deciding they are no longer fit to practise), pressures to keep working, and their sense of safety from the perpetrator. HCP-survivors indicated that when the perpetrator was a doctor, it was particularly difficult to get support and justice. Support is crucial for leaving or ending relationships with abusive people: being or feeling unable to access it leaves HCP-survivors entrapped.

Evidence suggests HCP-survivors rarely sought support for the mental and physical health consequences of DVA. Research with HCPs in general, that is, unrelated to DVA, has indicated a moderate to high prevalence of mental health problems and burnout due to the nature of healthcare (Grover et al., 2019; López-López et al., 2019; Naidu et al., 2019; O’Connor et al., 2018). Uptake of appropriate support for mental health problems among HCPs in general is low (Edwards & Crisp, 2017; Grover et al., 2019; Khan et al., 2014; Naidu et al., 2019; Weibelzahl et al., 2021) due to various barriers. These include feeling a need to keep working despite being acutely ill and/or exhausted, stigma, feeling they do not meet the threshold for support, concerns about confidentiality and their practice license and structural challenges relating to time and inadequate sickness cover (Aaronson et al., 2018; Clough et al., 2019; Smith et al., 2016; Spiers et al., 2017; Weibelzahl et al., 2021). These barriers echo those found in our review. HCPs experiencing physical illness face similar barriers (Kay et al., 2008). Research has additionally shown that doctors may self-prescribe for health problems (Grover et al., 2019; Roberts & Kim, 2015). HCPs who are experiencing DVA-related health outcomes may prefer to treat themselves rather than seek help.

Personal experience also affected HCPs’ responses to patients who were experiencing DVA. Much of the evidence identified in this review highlights how personal experience can enhance HCPs’ responses, which parallels findings from other qualitative studies excluded from our review (e.g. with midwife-survivors, Mezey et al., 2003). However, the included studies also showed that supporting patient-survivors could be difficult for HCPs who had not recovered from their experiences. Similarly, the midwife-survivors in Mezey et al.'s study (2003) found supporting patients with DVA brought back memories and feelings related to their own experiences, yet they felt they lacked resources to manage these feelings.

### Implications to Research, Policy, and Practice

While our review has shown that HCP-survivors wanted workplaces to be supportive and flexible, with a guarantee of confidentiality and support formalised in policy, research has not yet explored whether HCP-survivors want tailored interventions, what these interventions should include, and the mode and location of delivery (e.g. workplace, online, community or a blend). Community-based advocacy and therapy are cost-effective and reduce the risk of harm and re-victimisation, improving safety, mental health, self-esteem, quality of life, use of community resources (e.g. mental health services) and social support (Ogbe et al., 2020; Rivas et al., 2015; Trabold et al., 2020). However, HCPs may feel unable to access these interventions. Evaluations of UK interventions comprising patient-facing hospital-based DVA advocates mention that HCPs sought the advocate’s support, but providing such support was outside the advocate’s remit (McGarry & Nairn, 2015; Safelives, 2016). Nevertheless, this shows promise for interventions comprising a point of contact for support.

Studies included in this review showed that senior staff wanted onsite and offsite support for employees, suggesting the need for a whole-organisation approach (McLindon et al., 2020; RCM, 2018). Research about workplace interventions for DVA (beyond healthcare settings) has shown moderate evidence that DVA-focused supervisor training increases both their knowledge and the provision of information and resources for survivor-colleagues (Adhia et al., 2019). We recommend implementation of DVA training for staff in supportive roles (e.g. human resources, occupational health), coupled with referral pathways to DVA agencies to whom survivors can be referred. Research from Canada, the US and the UK has additionally emphasised a need for legislative and policy changes; employee assistance programmes; paid DVA leave and trade union support (e.g. Giesbrecht, 2020a; Wibberley et al., 2018). We recommend that healthcare organisations provide these kinds of practical measures to improve safety and wellbeing. The UK’s National Health Service (NHS) has a staff DVA policy that health providers can adopt, but it neglects emotional support and/or advocacy. What’s more, in a survey of 241 NHS providers, only 68% had a local policy and just two mentioned all measures (BMA, 2019). Improvement is needed here. We also recommend that professional registration bodies receive DVA training and implement policies to clarify that DVA disclosures will not automatically harm professional registration.

Further research is needed to investigate the needs of, and effective interventions for, HCP-survivors across HICs and LMICs, and different groups within those settings (job roles, rural and urban settings, etc.), including solutions for removing the barriers they face to accessing support. Interventions should address the physical and mental health consequences of physical and non-physical DVA, effects on patient care, the mental health problems and trauma that can arise from the day-to-day work of providing healthcare, and the intersection of DVA with workplace violence. Although IPV is common, interventions should also address other forms of DVA. Key learnings from research addressing poor mental health among HCPs could be adapted and applied (e.g. Carrieri et al., 2018). Research is additionally needed to explore how to address DVA perpetration by HCPs, especially since HCP-survivors may face additional risk, and find it harder to escape from, perpetrators employed within the same organisation. Table 2 summarises implications to practice, policy and research.

**Table 2.** Implications to policy, practice and research.

1	The unique barriers HCPs face to seeking DVA support may leave them entrapped, since support is crucial for leaving or ending relationships with abusive people. HCP-specific interventions are thus needed, either in the community or workplace, and workplace support should be formalised in policy
2	Community-based advocacy and therapy have multiple safety and health-related benefits, but HCPs may feel unable to access these interventions
3	Senior healthcare staff want onsite and offsite support for employees, suggesting the need for a whole-organisation approach for any workplace-based interventions
4	Research about workplace DVA interventions (beyond healthcare settings) has shown DVA-focused supervisor training increases both their knowledge and the provision of information and resources for survivor-colleagues. Research has additionally emphasised a need for legislative and policy changes; employee assistance programmes; paid DVA leave and trade union support
5	Further research is needed to investigate the needs of and effective interventions for, HCP-survivors across different country settings, and different groups within those settings, including solutions for removing the barriers they face to accessing support. Interventions should address the health consequences of DVA, effects on patient care, the intersection of DVA with workplace violence, as well as the mental health problems and trauma that can arise from the day-to-day work of providing healthcare
6	Research is additionally needed to explore how to address DVA perpetration by HCPs, especially since HCP-survivors may face additional risk, and find it harder to escape, from a perpetrator employed within the same organisation

### Limitations

The included studies are limited through low response rates and the use of non-validated measures or measures not encompassing non-physical forms of violence. Many measures oversimplify DVA and fail to capture the range of abusive behaviours, the situational and gendered contexts of violence and the independent and cumulative effects of different types of violence. Therefore, the prevalence and impact of DVA is likely under-estimated (Ford-Gilboe et al., 2016). The quantitative sample size was comprised of over 25,000 people, mostly women, and was diverse in terms of race, ethnicity, language, nationality, geography and culture. However, not all studies explored the relationship between, for example, ethnicity and DVA. Moreover, only two studies included gender minorities and only two explored whether sexual orientation was related to DVA. The review itself relied on searches conducted in English, and so did not include articles indexed in databases in other languages. Moreover, two articles in Turkish were excluded. These decisions left experiences of HCPs in non-English speaking regions less well explored. Additionally, the studies were heterogeneous, so meta-analyses on risk markers and outcomes were not conducted. Although we excluded studies that measured child abuse specifically, the lifetime prevalence meta-analysis included two studies where the age of family violence was not asked, thus some experiences may have been in childhood. Finally, although the pooled prevalence figures are arguably high, data do not exist to allow direct comparison with global prevalence.

## Conclusion

This is the first systematic review and meta-analysis of current and lifetime DVA among HCPs. We found a high pooled lifetime prevalence of DVA victimisation, particularly among women, nurses and LMIC HCPs. Risk markers were similar to those in the general population, but specific aspects of being a HCP made this population uniquely vulnerable to DVA. Additionally, there was an overlap between being a victim of DVA and experiencing workplace violence. DVA affected HCPs’ work, with staff being less able to concentrate and feeling too unwell to work. At the same time, HCP-survivors were more likely to identify and respond to DVA in patients, although, for HCPs with experience of recent DVA, this was distressing. Mental and physical health consequences were common, but HCPs rarely pursued support for these outcomes. Similarly, they rarely sought support for DVA, experienced unique barriers to doing so, and often found workplace support inadequate. Given the high prevalence of DVA, the potential impact on patients, HCPs’ experiences of burnout, mental ill-health and workplace violence, and the barriers HCPs experience to accessing support, there is a clear need for interventions tailored for HCPs. This need has been made more urgent by the COVID-19 pandemic. The pressure and intensity of HCPs’ work has increased due to staff shortages (e.g. Centres for Disease Control and Prevention, 2021) and the effects of the virus on patients, and in parallel, DVA rates have escalated (e.g. Amnesty International, 2021; ONS, 2020). Future research should focus on developing interventions in consultation with survivors, drawing on evidence about effective support for HCPs experiencing poor mental health or burnout, and interventions developed for survivors in the general population.

## Supplemental Material

sj-pdf-1-tva-10.1177_15248380211061771 – Supplemental Material for Healthcare Professionals& Own Experiences of Domestic Violence and Abuse: A Meta-Analysis of Prevalence and Systematic Review of Risk Markers and Consequences Click here for additional data file. Supplemental Material, sj-pdf-1-tva-10.1177_15248380211061771 for Healthcare Professionals’ Own Experiences of Domestic Violence and Abuse: A Meta-Analysis of Prevalence and Systematic Review of Risk Markers and Consequences by Sandi Dheensa, Elizabeth McLindon, Chelsea Spencer, Stephanie Pereira, Satya Shresta, Elizabeth Emsley and Alison Gregory in Trauma, Violence, & Abuse
