# Supplementary material for: Healthcare Professionals’ Own Experiences of Domestic Violence and Abuse: A Meta-Analysis of Prevalence and Systematic Review of Risk Markers and Consequences
Source: Trauma Violence Abuse. 2022 Jan 3;24(3):1282–99. doi: 10.1177/15248380211061771 (PMC10240650; doi:10.1177/15248380211061771)

Appendix A
Study characteristics and results

Quantitative and mixed methods studies

Author, year, country, own experience main topic (Y/N)	Sampling method	Genders (mixed / women), total sample size, and sample size by gender*)	Response rate	Job role breakdown *	Topic DVA or IPV only & sub-types **	Measures (paper survey unless otherwise stated)	Lifetime prevalence, by gender (where reported) and by subtype	Current/past 12 months prevalence, by gender (where reported), and by subtype
Acquadro-Maran and Varetto Italy 2018 Y	Convenience	Mixed: 147 (96 women, 51 men)	46%	59 nurses 37 psychologists 22 physicians 14 health technicians 9 health care operators 6 no answer	DVA P, S, V	Non-validated survey question	100% (HCPs recruited by virtue of experiencing DVA): **Physical** 10.20% (15/147) (all women) **Sexual** 2.04% (3/147) (all women) **Verbal**: 100% (147/147)	n/a
Aguocha et al. 2018 Nigeria Y	Random	Women only: 392	Not reported	19 management 199 senior staff 174 junior staff	IPV P,S,E, VF	Pre-tested non-validated survey questions	55.61% (218/392)	48.72% (191/392) **Physical** 63.35% (121/191) **Sexual** 71.73% (137/191) **Emotional** 67.02% (128/191)

Formatted: Font: Not Bold

								**Verbal** 100%<br>**Financial** 52.88% (101/191)
Al-Modall et al. 2015 Jordan<br>Y	Convenien ce	Women only: 147	Not reported	Nurses	IPV G	Not stated	100% (HCPs recruited by virtue of experiencing DVA)	n/a
Al Natour Gillespie, Wang et al. 2014 Jordan<br>N	Stratified random	Women only: 125	Not reported	39 midwives<br>69 staff nurses<br>17 diploma nurses	IPV: P, Ps, S	Women Abuse Screening Tool validated for Jordanian setting and non-validated survey questions	73.6% (92/125)	
Al-Natour, Gillespie, Felbinger et al. 2014 Jordan<br>Y	As above	Women only: 80 (subsampl e of the above)	60%	As above	As above	As above	Reported by subtype only<br>**Physical** 12.5% (10/80)<br>**Sexual** 5% (4/80)<br>**Emotional** 58.97% (46/78)	
Bacchus et al. 2006 UK<br>N	Not reported	Women only: 68	28.6% maternit y 38.7% GUM	56 maternity staff<br>12 GUM staff	IPV: P, S, Ps	Non-validated survey questions	33.82% (23/68)	

Baker USA 1998<br>N	Convenience	Women only: 87	28.5%	Emergency department nurses	IPV: P	Non-validated survey questions	24.14% (21/87)	2.3% (2/87)
Bracken et al. 2010<br>USA<br>Y	Convenience	Women only: 1981	52%	1504 nurses and 477 nursing personnel (some HCPs some not, not parsed)	IPV: P, S, E (includes SH)	Non-validated survey questions (online and paper)	30.74% (609/1981)<br>**Physical** 19.08% (378/1981)<br>**Sexual** 7.67% (152/1981)<br>**Emotional** 22.87% (453/1981)<br>**Physical** or **Sexual** 25.24% (500/1981)	Reported by subtype only<br>**Physical** 1.51% (30/1981)<br>**Sexual** 0.56% (11/1981)<br>**Emotional** abuse or **Sexual harassment** 2.42% (48/1981)
Çakmak Pekşen & Şahin 2019<br>Turkey<br>Y	Convenience	Mixed: 296 (103 women, 193 men)	96.7%	Physicians comprising 122 residents 117 specialists 57 general practitioners	DVA: P, S, E, V, F	Non-validated survey questions	41.55% (123/296)<br>**Physical** 4.39% (13/296)<br>**Sexual** 0.68% (2/296)<br>**Emotional** 33.78% (100/296)<br>**Verbal** 31.08% (92/296)<br>**Financial** 10.47% (31/296)	
Candib et al. 2012<br>USA<br>Y	Convenience	Mixed: 295 (151 women, 144 men)	45.5%	Physicians in family practice	IPV: P	Piloted non-validated survey questions derived from previously published studies	7.12% (21/295)	

Carlise-Cohen 1996<br>USA	Convenience	Women only (assumed): 80	77.6%	Members of women's health, obstetric and neonatal nurses' network	IPV: P, E	Piloted non-validated survey questions	38.75% (31/80)<br>**Emotional** 14% (11/80)<br>**Physical + Emotional** 25% (20/80)	
Carmona-Torres et al. 2018<br>Spain<br>Y	Random	Mixed: 1071 (794 women 277 men)	Not reported	534 physicians<br>502 nurses<br>35 nursing assistants	IPV: P, S, Ps, V	Two questionnaires used in previous study (different authors): one from Spain and one from USA but adapted for Spanish-speaking population	26.61% (285/1071)<br><br>**By gender**<br>34.01% (270/794) of women, 2.89% (8/277) of men	
Cavell Trust 2016<br>UK<br>N	Convenience	Mixed: 2254 (2067 women 129 men, 58 unspecified)	<1%	1540 nurses<br>207 midwives<br>435 healthcare assistants<br>72 nursing/nurse-related roles	IPV, P, NP	Questions taken from the IPV and partner abuse module of Crime Survey for England & Wales (online)	n/a	13.40% (302/2254)<br>**Physical** 3.1% (51/1646)<br>**Non-physical** 12.21% (201/1646)<br>Threats: 4.37% (72/1646)<br><br>**By gender:**<br>13.9% of women (215/1546) and 16%

Formatted: Font: Bold

								of men (16/100)
Christofides & Silo 2005<br>South Africa<br>Y	Convenience	Women only: 212	52%	Nurses	IPV: P, E	WHO multi-country study questionnaire (face-to-face)	39.15% (83/212)<br>**Physical** 14.62% (31/212)<br>**Emotional** 37.7% (80/212)	
deLahunter et al. 1996<br>USA<br>Y	Convenience	Mixed: 559 (177 women, 381 men, 1 unspecified)	59%	Physicians	DVA: P, S	Piloted non-validated survey questions plus two questions from Abuse Assessment Screen	9.48% (53/559)<br>**Physical** 7% (39/559)<br>**Sexual** 3.9% (22/559)<br><br>**By gender**<br>19.77% of women (35/177) and 4.72% of men (18/381)	
Diaz Olavarrieta et al. 2001<br>Mexico<br>Y	Convenience	Women only: 1150	90%	867 nurses, 283 nurses' aides	IPV: P, S, E	Questionnaire validated in previous study, translated, back translated, and adapted for Mexican context	Reported by subtype only<br>**Physical** 6.61% (76/1150)<br>**Sexual** 9.30% (107/1150)<br>**Emotional** 40% (460/1150)<br>**Physical** or **Sexual** 14.26% (164/1150)	

Donnelly et al. 2016<br>USA<br>Y	Convenience	Mixed: 400 (101 women, 296 men, 3 trans)	Not reported	110 emergency medicine technicians,<br>284 paramedics<br>7 'other' emergency medicine services personnel<br>2 unreported*	DVA: G	Non-validated survey questions (online)	16.5% (66/400)<br><br>**By gender**<br>39.60% of women (40/101)<br>8.11% of (n=24/296)
Doyle et al. 1999<br>USA<br>Y	Stratified random	Women only: 4501	59%	Physicians from a range of specialties	DVA: G	Non-validated survey question	3.69% (166/4501)
Early & Williams 2002<br>USA<br>Y	Convenience	Mixed: 195 (160 women, 35 men)	Not reported	Emergency nurses	IPV: P	Non-validated survey question	40% (78/195)
Elliott et al. 2002<br>USA<br>N	Convenience	Mixed: 1075 (276 women, 872 men)*	53%	Physicians (general internists, family practitioners, obstetrics/gynaecology [ob/gyn], emergency medicine)	DVA: D	Non-validated survey questions	13.58% (146/1075)<br><br>**By gender**<br>9.19% of men (n=76/827)<br>25.36% of women (n=70/276)

Guzmán-Rodríguez et al. 2019 Mexico Y	Not reported	Women only: 470	74%	Health workers	IPV: P, S, Ps	Adapted Conflict Tactics Scale and Index of Spousal Abuse, validated for use in Mexico	n/a	At time 2: 39.57% (186/470) **Physical** 16.17% (76/460) **Sexual** 13.19% (62/470) **Psychological** 35.53% (167/470)
Hinderliter 2000 USA N	Systematic randomised + stratified	Mixed: 557 (552 women, 4 men, 1 unstated)	34.6%	Nurses: mostly ob/gyn, followed by primary care, with some from paediatrics, geriatrics, psychiatry	IPV: P, G	Piloted non-validated survey questions	20.83% (116/557)	0.18% (1/557)
Janssen et al. 1998 Canada Y	Convenience	Women only: 198	99.5%	Obstetric nurses	IPV: P, S, E, C	Abuse Assessment Screen	37.88% (75/198) **Physical** 14.65% (29/198) **Sexual** 8.08% (16/198) **Emotional** 26.77% (53/198) **Control** 27.27% (54/198)	

Khan et al. 2015<br>Pakistan<br>Y	Convenience	Women only: 350	75%	185 nurses<br>165 doctors	DVA: P, S, E, V	Truncated version of WHO multi-country study	97.71% (342/350)<br>**Physical** 58% (203/350)<br>**Sexual** 55.14% (193/350) from husband, 10.29% (36/350) from in laws<br>**Emotional** 61.14% (214/350)<br>**Verbal** 96.1% (332/345)<br>**Controlling** behaviours reported by subtype with prevalence range of 51.9% - 69.2%
Kim & Motsei 2002<br>South Africa<br>N	Convenience	Women only: 36	Not reported	Nurses	IPV: P, S, E	Non-validated survey question plus focus group	69.44% (25/36)<br>**Physical** 27.78% (10/36)<br>**Sexual** 30.56% (11/36)<br>**Emotional** 66.67% (24/36)
La Flair et al. 2012<br>USA<br>Y	Random	Women only: 1438	52%	Healthcare workers	IPV: P, S, E, SH	Adaptation of the Abuse Assessment Screen<br><br>(10% online)	7.37% (106/1438)

Lai 2007<br>Taiwan<br>N	Unclear	Mixed: 252 (239 women, 13 men)	69.2%	Emergency room triage nurses	DVA: G	Pre-tested and piloted non-validated survey questions	3.17% (8/252)	
Mariano 2001<br>USA<br>N	Convenience	Women only: 70	34.4%	Nurses	IPV: P, NP	Partner Abuse Scales (**Physical** and non-**Physical**)	Reported by subtype only<br>**Physical** 7.14% (5/70)<br>**Non-physical** 15.71% (11/70)	
McLindon et al. 2018<br>Australia<br>Y	Convenience	Women only: 469 women	45%	172 midwives<br>145 nurses<br>69 doctors<br>61 allied HCPs (e.g., physiotherapists)<br>23 other*	DVA: P, S, E<br><br>May include child-hood abuse	IPV: Composite Abuse Scale<br><br>Family violence: piloted non-validated survey questions (paper and online)	39.02% (183/469) (212 minus 29 who witnessed IPV in childhood only)<br>**Severe Combined Abuse**, 13.78% (58/421)<br>**Physical** only 3.33% (14/421)<br>**Sexual** 12.11% (51/421)<br>**Emotional** +/or **harassment** only 8.08% (34/421)<br>**Physical** + **Emotional/harassment** 4.51% (19/421)	IPV only<br><br>11.52% (50/434)<br>**Severe Combined Abuse** 2.13% (8/375),<br>**Physical** only 0.53% (2/375)<br>**Sexual** only 1.87% (7/375)<br>**Emotional** abuse and/or **harassment** (6.67%, 25/375)<br>**Physical** +**Emotional** /**harassment** 2.13% (8/375)

McLindon et al. 2019<br>Australia<br>Y	Convenience	As above	As above	As above	As above	As above	As above	As above
Mitchell et al. 2013<br>Guyana<br>Y	Convenience	Mixed: 357 (294 women, 45 men, 18 unspecified)	87.4%	283 nurses, 50 physicians 30 unspecified*	IPV: P, E	Abuse Assessment Screen piloted in Guyanese context (victimisation) and 1 question modified from Demographic Health Survey (perpetration)	48.88% (174/356)<br><br>**By gender**<br>55.4% of women (163/294)<br>36% of men (16/45)<br>72.2% of unspecified (13/18)	8.96% (32/357)<br><br>8.16% of women (24/294)<br><br>8.88% of men (4/45)
Oliveira & d'Oliveira 2008<br>Brazil<br>Y	Unclear	Women only: 179	Not reported	50 nurses, 129 nursing aides/nurse technicians	DVA: P, S, Ps	WHO multi-country study questionnaire (face-to-face)	75.42% (135/179)<br><br>**Physical** IPV 33.52% (60/179) violence by relatives 23.46% (42/179) **Sexual** IPV 27.93% (50/179) violence by relatives 9.5% (17/179) – **Psychological** IPV 59.67% (105/176- missing data) **Violence by relatives** 30.17% (54/179)	Reported by subtype only<br>**Physical** IPV 2.79% (5/179) violence by relatives 3.91% (7/179) **Sexual** IPV 2.2% (4/179) violence by relatives nil **Psychological** IPV 12.85% (23/179) **Violence by relatives** 11.7% (21/179)

Formatted: Font: Not Bold

Ortlepp & Nkosi 1993 South Africa Y	Unclear	Women only: 65	Not reported	Nurses	IPV: P, NP	Index of Spouse Abuse piloted in South African context	92.31% (60/65) **Physical** 92.31% (60/65) **Non-physical** 92.31% (60/65)	
Price 1997 USA	Convenien ce	Women only: 314	32%	Nurses	IPV: P	Non-validated survey questions	14.97% (47/314)	0.96% (3/314)
Ralph 2000 USA	Convenien ce	Women only: 46	74%	Ob/gyn nurses	IPV: P	Non-validated survey question	4.35% (2/46)	21.74% (10/46)
Rappleyea et al. 2009 USA	Convenien ce	Mixed: 84 (58 women, 26 men)	11.2%	Mental health professionals	IPV	Not reported	17.86% (15/84)	
RCM 2018 UK Y	Convenien ce	Women only: 229	Not reported	240 midwives 9 maternity support workers*	DVA: D	Non-validated survey question (online) with free text options	81.66% (187/229)	
Reibling et al. 2020 USA Y	Convenien ce	Mixed: 400 (175 women, 225 men)	Not reported	Physicians	IPV: P, S, Ps, C, St	Piloted non-validated survey questions derived from literature (online)	n/a	24% (96/400) **Physical** 7.5% (30/400) **Sexual** 4% (16/400) **By gender:** 28.57% (50/175) women, 20.44% (46/225) men

Rodriguez et al. 1999<br>USA<br>N	Stratified probability (random)	Mixed: 400 (168 women, 232 men-estimated)	69%	Physicians:<br>149 family medicine<br>115 internal medicine<br>136 ob/gyn	IPV: P	Piloted non-validated survey questions	12% (48/400)	
Sawyer et al. 2018<br>Australia<br>N	Convenience	Mixed: 28 (10 women, 18 men)	16.4%	Paramedics	IPV: G	Non-validated survey questions (online)	39.29% (11/28)<br>**By gender**<br>60.00% (6/10) of women<br>27.78 (5/18) of men	
Selek et al. 2012<br>Turkey<br>Y	Convenience	Women only: 96	Not reported	Nurses	DVA: P, S, V, F	Abuse Assessment Screen adapted for use in Turkish context	22.92% (22/96)<br>**Physical** 4.17% (4/96)<br>**Sexual** 4.17% (4/96)<br>**Verbal** 4.17% (4/96)<br>**Financial** 10.42% (10/96)<br>More than one type 3.13% (3/96)	
Sharma and Vatsa 2011<br>India<br>Y	Convenience	Women only: 60	5.2%	Nurses	IPV: P, S, E, C	WHO multi-country study questionnaire	75% (45/60)<br>**Physical** 43.33% (26/60)<br>**Sexual** 30% (18/60)<br>**Emotional/psychological** 65.% (39/60)<br>**Control** 60% (36/60)<br>**Physical** + **Sexual** 23% (14/60)	Reported by subtype only<br>**Physical** 35% (21/60)<br>**Sexual** 16.67% (10/60)<br>**Emotional/psychological** 48.33% (29/60)

Shokre & Ahmed 2017 Egypt Y	Consecutive	Women only: 324	Not reported	85 head nurses 239 staff nurses	IPV: P, Ps	Women Abuse Screening Tool and Women's Experience with Battering scale plus some piloted non-validated survey questions	59.57% (193/324)
Siltala et al. 2019 Finland Y	Convenience	Mixed: 1951 (1683 women and 268 men)	54%	131 doctors 1102 nurses 719 other*	DVA: P, S, Ps<br><br>May include child-hood abuse	Non-validated survey question (online)	37.98% (741/1951)<br>**Physical** only 1.79% (35/1951)<br>**Sexual** only 0.36% (7/1951)<br>**Psychological** only 18.5% (361/1951)<br>**Physical** & **Sexual** 0.1% (2/1951)<br>**Physical** + psych 12.3% (240/1951)<br>**Physical** + **Sexual** + **Psych** 3.59% (70/1951)<br>**-Sexual** + **Psych** 1.17% (23/1951)

Stein et al. 2021 USA Y	Convenience	Mixed: 882 (616 women, 264 men, 1 non-binary, 1 unspecified)	Not calculable	Surgeons	IPV: P, S, E, C	Validated survey developed by co-author for previous study	60.77% (536/882) **Physical** 13.15% (116/882) **Sexual** 9.64% (85/882) **Emotional** 57% 507/882 **Control**: 36% (316/882)	
Stenson & Heimer 2008 Sweden Y	Random	Women only: 588	68%	59 paramedic staff 41 physicians 270 nurses /midwives 218 staff nurses	IPV: P, S, E	Norvold Abuse Questionnaire plus piloted non-validated survey questions	23.47% (138/588) **Physical** 16.5% (97/588) **Sexual** 6.46% (38/588) **Emotional** 13.95% (82/588)	
Sundborg et al. 2012 Sweden N	Convenience sample of participants in randomly selected sites	Mixed: 191 (190 women, 1 man)	69.3%	Nurses	IPV: G	Piloted non-validated survey questions	12.04% (23/191)	
Weiss et al. 1999 USA Y	Convenience	Mixed: 49 (10 women, 36 men, 3 unspecified)	26.3%	Emergency medicine technicians	IPV: P, NP	Index of Spousal Abuse and non-validated self-report questions	26.53% (13/49) (according to self-report)	2.04% (1/49) (according to self-report)

Qualitative studies

**Author, year, country, own experience main topic (Y)**	**Total (all survivors were women)**	**Job role**	**IPV/ DVA**
Donovan et al.<br>2020<br>UK<br>Y	21	Doctors	DVA
McLindon et al.<br>2020<br>Australia<br>Y	93 (plus 18 interviews with managers)	Mostly nurses and midwives	DVA
Sprague et al.<br>2015<br>South Africa<br>Y	20	Nurses	IPV

Note. * Ns do not add up to total sample N because some participants did not answer the question about personal DVA but authors did not give a gender or job role breakdown of respondents.

Note. **P=Physical, S=Sexual, Ps=Psychological, V=Verbal, F=Financial, N=neglect, NP=Non-physical, SH=Sexual harassment, St=Stalking, C=Control, G=general question (e.g., have you experienced DVA/ does your partner make you feel afraid), D=question that gave comprehensive definition.

Appendix B
Risk of Bias

Author	1. Was the study's target population a close representation of the national population in relation to relevant variables?	2. Was the sampling frame a true or close representation of the target population?	3. Was some form of random selection used to select the sample?	4. Was the likelihood of non-response bias minimal (i.e. 65%+)?	5. Were data collected directly from the participants (as opposed to a proxy)?	6. Was an acceptable case definition used in the study (i.e., did DVA definition include nonphysical forms)?	7. Was the study instrument that measured DVA shown to have reliability and validity?	8. Was the same mode of data collection used for all participants?	9. Was the length of the shortest prevalence period for DVA experience appropriate?	10. Was numerator & denominator for DVA appropriate?	TOTAL
Acquadro			1	1		1	1				4
Aguocha				1			1				2
Al-Modall			1	1		1	1				4
Al Natour				1							1
Al-Natour				1							1
Bacchus			1	1			1				3
Baker			1	1		1	1				4
Bracken			1	1			1	1			4
Çakmak			1				1				2
Candib			1	1		1	1				4
Carlise-Cohen			1				1				2
Carmona-Torres				1							1
Cavell			1	1							2
Christofides			1	1							2
Delaunter			1	1		1	1				4
Diaz			1								1
Donnelly			1	1			1				3
Doyle							1				1
Early			1	1		1	1				4
Elliott			1	0			1				2
Guzmán-			1								1
Hinderliter				1			1				2
Janssen			1								1

Appendix B
Risk of Bias

Author	1. Was the study's target population a close representation of the national population in relation to relevant variables?	2. Was the sampling frame a true or close representation of the target population?	3. Was some form of random selection used to select the sample?	4. Was the likelihood of non-response bias minimal (i.e. 65%+)?	5. Were data collected directly from the participants (as opposed to a proxy)?	6. Was an acceptable case definition used in the study (i.e., did DVA definition include nonphysical forms)?	7. Was the study instrument that measured DVA shown to have reliability and validity?	8. Was the same mode of data collection used for all participants?	9. Was the length of the shortest prevalence period for DVA experience appropriate?	10. Was numerator & denominator for DVA appropriate?	TOTAL
Khan			1								1
Kim			1	1			1				3
La Flair				1				1			2
Lai			1				1				2
Mariano			1	1							2
McLindon			1	1				1			3
McLindon			1	1				1			3
Mitchell			1								1
Oliveira			1	1							2
Ortlepp			1	1							2
Price			1	1		1	1				4
Ralph			1	1		1	1				4
Rappleyea			1	1			1				3
RCM			1	1			1				3
Reibling			1	1			1				3
Rodriguez						1	1				2
Sawyer			1	1			1				3
Selek			1	1							2
Sharma			1	1							2
Shokre			1	1							2

Appendix B
Risk of Bias

Author	1. Was the study's target population a close representation of the national population in relation to relevant variables?	2. Was the sampling frame a true or close representation of the target population?	3. Was some form of random selection used to select the sample?	4. Was the likelihood of non-response bias minimal (i.e. 65%+)?	5. Were data collected directly from the participants (as opposed to a proxy)?	6. Was an acceptable case definition used in the study (i.e., did DVA definition include nonphysical forms)?	7. Was the study instrument that measured DVA shown to have reliability and validity?	8. Was the same mode of data collection used for all participants?	9. Was the length of the shortest prevalence period for DVA experience appropriate?	10. Was numerator & denominator for DVA appropriate?	TOTAL
Siltala			1	1			1				3
Stein			1	1			1				3
Stenson											0
Sundborg			1				1				2
Weiss			1	1							2

Appendix C

Name	Gender	Sample Type	Statistics for each study<br>Event rate	Lower limit	Upper limit
Aguocha et al.	W	Mixed	0.556	0.507	0.605
Al Natour et al.	W	Nurses and midwives	0.736	0.652	0.806
Bacchus et al.	W	Mixed	0.338	0.236	0.458
Baker	W	Nurses	0.241	0.163	0.342
Bracken et al.	W	Nurses	0.307	0.287	0.328
Çakmak-Peksen & Sahin	Combined	Physicians	0.416	0.361	0.473
Candib et al.	Combined	Physicians	0.071	0.047	0.107
Carlise-Cohen	W	Nurses	0.388	0.287	0.498
Carmona-Torres et al.	M	Mixed	0.029	0.015	0.057
Carmona-Torres et al.	W	Mixed	0.340	0.308	0.374
Christofides & Silo	W	Nurses	0.392	0.328	0.459
Delahunter	M	Physicians	0.047	0.030	0.074
Delahunter	W	Physicians	0.198	0.145	0.263
Donnelly et al.	M	Mixed	0.081	0.055	0.118
Donnelly et al.	W	Mixed	0.198	0.131	0.287
Doyle et al.	W	Physicians	0.037	0.032	0.043
Early & Williams	Combined	Nurses	0.400	0.334	0.470
Elliott et al.	M	Physicians	0.092	0.074	0.114
Elliott et al.	W	Physicians	0.254	0.206	0.308
Hinderliter	Combined	Nurses	0.208	0.177	0.244
Janssen et al.	W	Nurses	0.379	0.314	0.448
Khan et al.	W	Mixed	0.977	0.955	0.989
Kim et al.	W	Nurses	0.694	0.528	0.822
La Flair et al.	W	Mixed	0.074	0.061	0.088
Lai	Combined	Nurses	0.032	0.016	0.062
McLindon et al.	W	Mixed	0.390	0.347	0.435
Mitchell et al.	M	Mixed	0.489	0.437	0.541
Mitchell et al.	W	Mixed	0.289	0.176	0.436
Oliveira & d'Oliveira	W	Nurses	0.754	0.686	0.812
Ortlepp & Nkosi	W	Nurses	0.923	0.828	0.968
Price	W	Nurses	0.150	0.114	0.194
Ralph	W	Nurses	0.043	0.011	0.158
RCM	W	Midwives	0.817	0.761	0.862
Rodriguez et al.	Combined	Physicians	0.120	0.092	0.156
Sawyer et al.	M	Paramedics	0.278	0.121	0.519
Sawyer et al.	W	Paramedics	0.600	0.297	0.842
Selek et al.	W	Nurses	0.229	0.156	0.324
Sharma & Vatsa	W	Nurses	0.750	0.626	0.843
Shokre & Ahmed	W	Nurses	0.596	0.541	0.648
Siltala et al.	Combined	Mixed	0.380	0.359	0.402
Stein et al.	M	Surgeons	0.481	0.421	0.541
Stein et al.	W	Surgeons	0.644	0.606	0.681
Stenson & Heimer	W	Mixed	0.235	0.202	0.271
Sundborg et al.	Combined	Nurses	0.120	0.081	0.175
Weiss et al.	Combined	Emergency medicine technician	0.265	0.161	0.405
			0.309	0.302	0.317

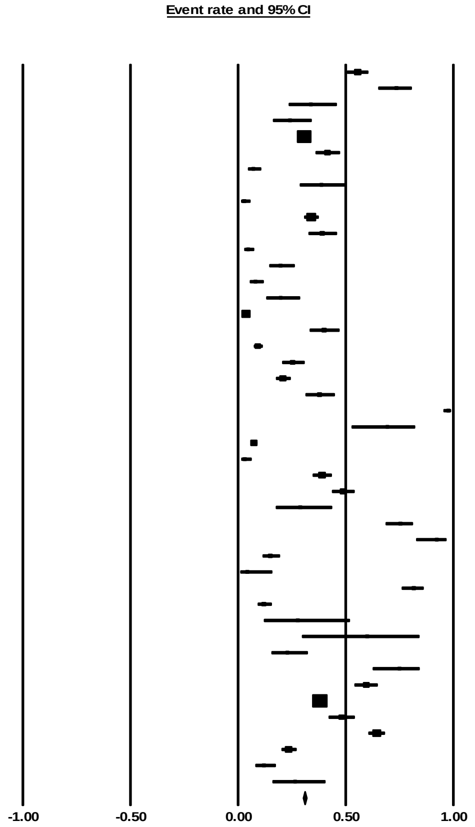

Forest plot for lifetime prevalence of domestic violence and abuse (DVA) victimisation.

Forest plot for past-year prevalence of domestic violence and abuse (DVA) victimisation

Appendix D

Risk markers and outcomes for domestic violence and abuse (DVA) among healthcare professionals (HCPs)

Author	Result	Statistic as reported in the study
	**RISK MARKERS**	
**Gender**		
Siltala et al. (2019)	i) The percentage of women was lower than the percentage of men in the 'no violence experienced' group.<br><br>The percentage of women was higher than the percentage of men in the following violence groups:<br>ii) psychological<br>iii) psychological & physical<br>iv) psychological, physical & sexual.	$\chi2$ (7, N=1951) = 40.31, p < 0.001)<br><br>i. 59.5%, 95% CI [57.2, 61.8] vs 78.7%, 95% CI [73.8, 83.6]<br>ii. 19.4%, 95% CI [17.5, 21.3] vs 12.7%, 95% CI [8.7, 16.7]<br>iii. 13.1%, 95% CI [11.5, 14.7] vs 7.1%, 95% CI [4.0, 10.2]<br>iv. 4.2%, 95% CI [3.32, 5.2] vs 0.4%, 95% CI [0.4, 1.2]*
Candib et	Being a woman was associated with experience of DVA.	$\chi2$ = 10.8, p =.001

al. (2012)		42.4%, 95% CI [34.5, 50.3] vs 24.3% 95% CI [17.3, 31.3]
Donnelly et al. (2016)		χ2. (4, n=397) = 60.25, p<.001)*<br>40% vs 8%
Rodriguez et al. (1999)		20% vs 10%, p=.01
**Ethnicity**		
Reibling et al. (2020)	Being Asian American was associated with higher rates of DVA than White, Hispanic, or other ethnicities.	χ^2= 11.6, p =.001 (31.7% vs 25.4% vs 16.7% vs 10.4%)
Bracken et al. (2010)	Asian women had 55% lower odds of reporting physical/sexual violence, threats, and stalking.<br><br>Asian women had 69% lower odds of reporting emotional abuse/sexual harassment, while being Latina or White increased odds of emotional abuse/sexual harassment.	x2 = 214.17, p < .001,<br>OR= 0.45, 95% CI [0.27, 0.76]<br><br>x2 = 227.50, p < .001:<br>Asian OR= 0.31, 95% CI [0.15, 0.67]<br>Latina OR= 2.11, 95% CI [1.01, 4.43]<br>White OR=1.75, 95% CI [1.32, 2.31]
Doyle et al. (1999).	Being White was associated with higher odds of reporting DVA.	OR = 6.21, 95% CI [1.58, 24.46]
**Number of children**		
Shokre & Ahmed (2017)	Number of children and experience of DVA was positively correlated.	rs= -0.117, p<.05
Aguocha et al. (2017)	Number of children was associated with experience of DVA where women with 1-4 children had 70% lower odds of experiencing DVA than those with 0 children. Those who had 5+ children had higher odds (data not shown).	x² = 17.9, p < .001,<br>OR = 0.3, 95% CI [0.2, 0.6]
**Income/expenditure**		
Carmona-Torres et al. (2018)	Source of household income was associated with experience of DVA (women who were reliant on a ‘combined income’ i.e., their own and their partner’s income, had 53% lower odds of	OR= 0.481, 95% CI [0.311, 0.743]

	experiencing DVA than those reliant on their own salary).	
Sharma & Vatsa (2011)	Physical violence was higher if husband was unemployed compared with unskilled, semi-skilled, or professional.	66.7% vs 51.7% vs 0% vs 20%, p=.03
Shokre & Ahmed (2017)	Greater share in household expenses was positively correlated with higher score on a DVA scale.	rs = 0.333, p<0.01
**Education**		
Aguocha et al. (2017)	Education was associated with DVA: women with tertiary education had 54% reduced odds of DVA than those with secondary or less.	$X^2 = 8.9$, $p < .001$,<br>OR=0.46, 95% CI [0.28, 0.8]
Khan et al., (2014)	Those with an undergraduate or graduate degree were less likely to experience emotional abuse than those with a diploma.	p=.028
Sharma & Vatsa (2011)	Sexual violence was higher if HCP had lower qualification (lowest qualification vs mid vs graduate).	100% (n=2) vs 35.6% (n=16) vs 0% (n=0)<br>p=.02
Bracken et al. (2010)	Education was associated with physical/sexual violence, threats, and stalking, where those with a college degree had 30% lower odds of experiencing these types of DVA.	x2 = 214.17, p < .001,<br>OR =0.70 (0.54,0.92)
	**OUTCOMES (could also be RISK MARKERS)**	
**Mental health**		
Sharma & Vatsa (2011)	Over half of the study subjects (56.7%) reported that physical or sexual violence affected their physical and mental health.	
Doyle et al. (1999)	HCPs with personal experience of DVA had more days of poor mental health in the last month than those who had not experienced DVA, and had greater odds of depression histories, suicide attempt histories, and severe stress at home compared with HCPs without experience.	Days of poor mental health: 0 days 2% (p<.001), 1 or 2 days 3.8%, >3 days 6.2%<br>Depression: OR=3.21, 95% CI [2.04, 5.03], p<.001<br>Suicide attempt: OR=2.47, 95% CI [1.03, 5.90], p<.05<br>Severe stress: OR=2.55, 95% CI [1.27, 5.13], p<.01
Guzmán-	Depression in HCPs experiencing DVA was higher than HCPs	aOR=2.9, 95% CI [1.4, 6.2]

Rodríguez et al. (2019)	without experience.	Physical aOR=4.3, 95% CI [1.8, 10.1]<br>Verbal/psychological aOR=3.1, 95% CI [1.4, 6.6]<br>Sexual aOR=3.1, 95% CI [1.2, 8.2]
La Flair et al. (2012)	Women experiencing recent DVA had increased depression scores.	OR=1.39; 95% CI [0.87, 1.91] $p < .001$
Weiss et al. (1999)	An association was found between experiencing DVA and having a psychiatric history	Psychiatric history RR 7.00, 95% CI [2.1, 23.9]
Stein et al. (2021)	Surgeons with a personal history of mental illness had 2.3x greater odds of experiencing DVA ($p<.0001$).	OR=2.32, $p <0.001$

Note. *additional statistical data provided by authors

Name	Gender	Sample Type	Event rate	Lower limit	Upper limit
Aguocha et al.	W	Mixed	0.487	0.438	0.537
Guzmán-Rodríguez et al.	W	Mixed	0.396	0.352	0.441
Reibling et al.	Combined	Physicians	0.242	0.203	0.287
Ralph	W	Nurses	0.217	0.121	0.359
Cavell Nurses Trust	X	Nurses and midwives	0.134	0.121	0.149
McLindon et al.	W	Mixed	0.115	0.088	0.149
Mitchell et al.	Combined	Mixed	0.083	0.058	0.117
Baker	W	Nurses	0.023	0.006	0.087
Weiss et al.	Combined	Emergency medicine technician	0.020	0.003	0.131
Price	W	Nurses	0.010	0.003	0.029
Hinderliter	X	Nurses	0.002	0.000	0.013
			0.214	0.202	0.228

Event rate and 95% CI

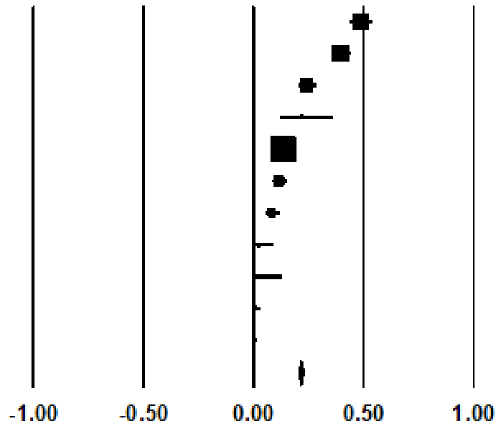

Supplement: sj-pdf-1-tva-10.1177_15248380211061771 – Supplemental Material for Healthcare Professionals& Own Experiences of Domestic Violence and Abuse: A Meta-Analysis of Prevalence and Systematic Review of Risk Markers and Consequences [file sj-pdf-1-tva-10.1177_15248380211061771.pdf]
